# The TNFA -857C/T Polymorphism: Association with Rheumatoid Arthritis and Anti-CCP Levels in a Mexican Population

**DOI:** 10.1155/2019/2637607

**Published:** 2019-10-09

**Authors:** Juan Manuel Agraz-Cibrián, Gabriela Nohemí Espinoza-De León, Ma. de Jesús Durán-Avelar, Norberto Vibanco-Pérez, Liliana Ortiz-Martínez, Alejandro Vázquez-Reyes, Jorge Gutiérrez-Franco, Miriam Fabiola Ayón-Pérez, José Francisco Zambrano-Zaragoza

**Affiliations:** ^1^Unidad Académica de Ciencias Químico Biológicas y Farmacéuticas, Universidad Autónoma de Nayarit, Tepic, Nayarit, Mexico; ^2^Clínica de Reumatología, Servicio de Medicina Interna, Instituto Mexicano del Seguro Social HGZ No. 1, Tepic, Nayarit, Mexico

## Abstract

Rheumatoid arthritis (RA) is a chronic inflammatory disease whose association with SNPs has led to the identification of biomarkers in different populations. To determine the association of the -857C/T SNP of the *TNFA* gene with RA and clinical parameters, 233 RA patients and 237 healthy controls were included in this study. The -857C/T polymorphism was determined using the TaqMan® system and clinical features were also determined. We found that the -857C/T SNP was in Hardy-Weinberg equilibrium. Our results showed no association of the -857C/T SNP with RA; however, RA patients carrying the TT genotype showed lower anti-CCP levels than other groups. Therefore, the TT genotype could be a risk factor for developing anti-CCP-negative RA. Our results suggest that the T allele of the *TNFA* -857C/T SNP exerts an influence on anti-CCP levels and could be a candidate marker for anti-CCP-negative RA.

## 1. Introduction

Rheumatoid arthritis (RA) is a chronic inflammatory disease that affects joints, with a prevalence of approximately 1% in the worldwide population, [1] but with an overall prevalence in Mexico estimated at 1.6% [2]. Women are affected more often than men, in a proportion of 3 : 1 [1]. RA is characterized by the production of two known antibodies, called rheumatoid factor (RF) and anticyclic citrullinated peptide antibodies (CCP) [3]. It has been reported that the anti-CCP antibodies are present in about two-thirds of RA patients [4]. Therefore, RA can be considered to contain two separate subsets: anti-CCP-positive and anti-CCP-negative, each with distinct genetic and environmental risk factors [5].

RA is an autoimmune disease that involves both environmental and genetic factors. The impact of genetic factors compared to environmental ones is supported by the 15-30% concordance rates of RA in monozygotic twins. Moreover, it has been shown that up to 60% of disease susceptibility is due to genetic factors [6], including the polymorphisms in genes that encode proinflammatory cytokines, which can play an important role by amplifying the inflammatory events that this disease triggers [7].

Tumor necrosis factor alpha (TNF*α*) is a potent pleiotropic proinflammatory cytokine produced mainly by macrophages, though other cells, such as T, B, and NK cells can produce it as well [8]. Among the functions of TNF*α*, the secretion of other cytokines increases the expression of adhesion molecules in the endothelium and promotes neutrophil activation and migration. Costimulatory effects on T-cell activation and antibody production by B-lymphocytes have been implicated in RA pathogenesis [7]. In addition, RA patients with elevated levels of TFN*α* in sera and synovial fluid show greater articular damage [9].

The *TNFA* gene is located on the short arm of chromosome 6 at locus 6p21.3 in the MCH class III region [7]. Among the SNPs described in the promoter region of *TNFA*, the -857C/T SNP affects the transcription of the *TNFA* gene.

The -857T allele variant of the *TNFA* promoter contains a transcription factor OCT1 binding site (ATGAAGAC) from position -858 to position -851. OCT1 binds to the sequence only with the -857T allele, but not with the -857C allele, and then inhibits *TNFA* promoter and expression activity [10, 11].

In the present study, we determined the association of the *TNFA* -857C/T SNP with RA and the effect of the genotypes of this SNP on anti-CCP levels, DAS28, and sHAQ-DI in a group of RA patients from western Mexico.

## 2. Materials and Methods

### 2.1. Subjects

A total of 233 consecutive, unrelated RA patients, regardless of disease duration, participated in this study. All were diagnosed according to the ACR/EULAR 2010 criteria [12] at the IMSS General Hospital No. 1 in Tepic Nayarit, Mexico. The DAS28 (Disease Activity Scores using 28-joint counts) [13] and sHAQ-DI (Spanish version of the Health Assessment Questionnaire Disability Index) scores [14] were determined by an experienced rheumatologist. A total of 237 clinically healthy subjects were included as a control group. All participants were Mexican residents from the state of Nayarit who gave their informed consent prior to inclusion in the study, according to the 1964 Declaration of Helsinki and its later amendments [15]. The study was approved by the local ethics committee at the Instituto Mexicano del Seguro Social, Tepic, Nayarit (protocol number 1802, approved on 25 March 2013).

### 2.2. Genotyping the *TNFA* -857C/T SNP and Anticyclic Citrullinated Peptides Antibody (Anti-CCP) Levels

To genotype the *TNFA* -857C/T SNP, we used the predesigned SNP genotyping assay (part number: C__11918223_10, Foster City, CA, USA) provided by Applied Biosystems. To determine the anticyclic citrullinated peptides antibody (anti-CCP) levels by enzyme-linked immunosorbent assay (ELISA) (DRG, EIA-5653), we followed the methods outlined in Durán-Avelar et al. [16].

### 2.3. Statistical Analyses

All statistical analyses were done following the methods in Durán-Avelar et al. [16].

## 3. Results

As Table 1 shows, 91.85% of the RA patients were female and 73.4% of them were positive to anti-CCP. No significant differences in age or the female/male ratio were found between RA patients and controls.

Our results show that the *TNFA* -857C/T polymorphism was in Hardy-Weinberg equilibrium in both patients and controls (*p* = 0.31 and 0.81, respectively). However, no significant association of the -857C/T polymorphism has been found between RA patients (regardless of their anti-CCP status) and controls in any of the genetic models tested (Table 2).

To determine whether the -857C/T SNP has an effect on anti-CCP levels, the RA patients were divided into two groups—anti-CCP-positive or anti-CCP-negative—and the association with the -857C/T SNP was ascertained. The T allele could be a risk factor for developing anti-CCP-negative RA (codominant, recessive, and addictive models, OR = 3.8456, 3.5204, and 1.6923; 95% CI: 1.3028-11.3512, 1.2087-10.2535, and 1.0573-2.7086, respectively), although the large confidence intervals could be due to the small size of the TT genotype (Table 3). Hence, this finding requires further analysis with a larger sample of anti-CCP-negative RA patients.

**Figure 1:**
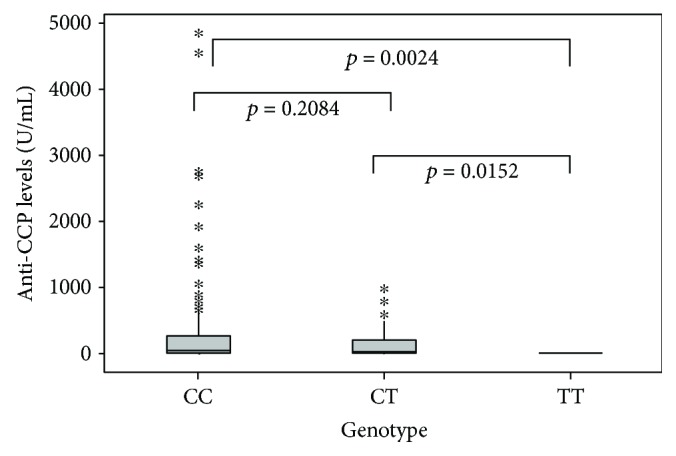
Anti-CCP levels in RA patients grouped according to their genotype, -857-C/T. Comparisons were performed using the Mann–Whitney *U* test.

Another finding was that the RA patients who carried the TT genotype showed lower levels of anti-CCP (Figure 1) than those with the CT (*p* = 0.0152) or CC genotypes (*p* = 0.0024). However, the DAS28 and sHAQ-DI scores showed no statistical differences between RA patients grouped according to their genotypes (ANOVA test, *p* = 0.813, and Kruskal-Wallis test, *p* = 0.746, respectively).

## 4. Discussion

The present study analyzed the association of the *TNFA* -857C/T SNP with RA. It determined that the *TNFA* -857C/T SNP is not associated with the risk of developing RA. Few studies of the association of this SNP with RA have been published, but our data agree with those reported for a Pakistani population [17], though not with those from a Chinese Han sample. It is important to note that the allelic frequencies in the study of the Chinese Han population were not in the Hardy-Weinberg equilibrium [18]. We further found that RA patients carrying the TT genotype showed lower anti-CCP levels than those with the CT or CC genotypes (Figure 1). As mentioned above, the T allele of this SNP affects *TNFA* expression, and it has been reported that blocking TNF*α* decreases anti-CCP levels [19], suggesting that TNF*α* is necessary for anti-CCP production. Moreover, B cells play a pivotal role in RA pathogenesis [20]. It has been reported that blocking TNF*α* does not affect the expression of CD154 on B cells, so the promotion of B and T cell proliferation, antibody formation, and immunoglobulin switching are not affected [19].

This is the first study to report the association of this SNP with anti-CCP levels, suggesting the role that this SNP plays in relation to clinical features of RA.

RA can also be understood as containing two separate subsets—anti-CCP-positive and anti-CCP-negative—each with distinct genetic and environmental risk factors [5]. The T allele of the -857C/T SNP could be a risk factor for developing the anti-CCP-negative RA (Table 3), but not the anti-CCP-positive RA (data not shown), though this affirmation requires further analysis with a larger sample of anti-CCP-negative RA to confirm the role of this SNP in this sub-type of RA. This fact could be explained by the finding that the putative lower production of TNF*α* [10] could provoke less inflammation, making early diagnoses difficult, since cases must show greater joint inflammation than anti-CCP-positive patients to be classified as RA, according to the 2010 criteria. The pathogenesis of this RA subset is less well understood than the anti-CCP-positive RA subset [5], though there are reports that anti-CCP-positive RA patients experience longer disease duration [21], higher acute-phase reactants, short-term radiographic progression [22], and a reduced response to anti-TNF treatment [23]. These features are likely due to lower TNF*α* production, because studies have found that TNF*α* is required for anti-CCP production [19] and that anti-CCP promotes local inflammation; therefore, they are related to the severity of RA. These data suggest that the behavior of the disease could differ in anti-CCP-positive vs. anti-CCP-negative patients. These results could contribute to proposing an additional marker for anti-CCP-negative RA.

Our results suggest that the T allele of the *TNFA* -857C/T SNP influences anti-CCP levels and could be a candidate marker for analysis as a risk factor for anti-CCP-negative RA.

## Figures and Tables

**Table 1:** Sociodemographic and clinical characteristics of RA patients and controls.

	RA patients		Controls
	Anti-CCP-positive	Anti-CCP-negative	
*n*	171	62	237
Age	53.04 ± 14.6	51.9 ± 12.02	49.9 ± 12.7
Female/male	158/13	56/6	218/19
DAS28^a^	4.33 ± 1.4	4.24 ± 1.4	—
Anti-CCP (U/mL)^a^	334.9 ± 665.25	4.4 ± 2.09	—
sHAQ-DI^b^	1.0 (0.0, 2.5)	1.0 (0.0, 2.0)	—
Treatment			—
Biologics (*n*)	32	4	—
Etanercept	22	3	—
Adalimumab	9	0	—
Tocilizumab	1	1	—
Methotrexate (*n*)	171	55	—
Corticosteroids (*n*)	155	50	—

RA: rheumatoid arthritis; anti-CCP: anticyclic citrullinated peptide antibodies; DAS28: Disease Activity Scores using 28-joint counts; sHAQ-DI: Spanish version of the Health Assessment Questionnaire Disability Index. ^a^Mean ± SD; ^b^median (min, max).

**Table 2:** Association of the *TNFA* -857C/T polymorphism in RA patients compared to controls.

Genetic model	Genotype	Frequencies		OR	95% CI	*p*
		RA (*n* = 233)	Controls (*n* = 237)			
Co	**CC**	166	166	1.0000	—	—
	**CT**	58	64	0.9063	(0.5980-1.3735)	0.642
	**TT**	9	7	1.2857	(0.4683-3.5302	0.625

Do	**CC**	147	166	0.9437	(0.6344-1.4038)	0.884
	**CT+TT**	61	71			

Re	**CC+CT**	224	230	1.3202	(0.4834-3.6056)	0.587
	**TT**	9	7			

Additive	**C**	390	396	1.3055	(0.814-3.5402)	0.952
	**T**	76	78			

RA: rheumatoid arthritis; CI: confidence interval; OR: odds ratio; Co: codominant; Do: dominant; Re: recessive.

**Table 3:** Association of the *TNFA* -857C/T polymorphism in RA patients with anti-CCP-negative status to controls.

Genetic model	Genotype	Frequencies		OR	95% CI	*p*
		RA (*n* = 62)	Controls (*n* = 237)			
Co	**CC**	37	166	1.0000	—	—
	**CT**	19	64	1.3319	0.7138-2.855	0.367
	**TT**	6	7	3.8456	1.3028-11.3512	0.014

Do	**CC**	37	166	1.5797	0.873-2.8126	0.120
	**CT + TT**	25	71			

Re	**CC + CT**	56	230	3.5204	1.2087-10.2535	0.0121
	**TT**	6	7			

Additive	**C**	93	396	1.6923	1.0573-2.7086	0.020
	**T**	31	78			

RA: rheumatoid arthritis; CI: confidence interval; OR: odds ratio; Co: codominant; Do: dominant; Re: recessive.

## Data Availability

The genotype, anti-CCP levels, DAS28, and HAQ values used to support the findings of this study are available from the corresponding author upon request.
